# Vibrotactile feedback as a countermeasure for spatial disorientation

**DOI:** 10.3389/fphys.2023.1249962

**Published:** 2023-11-03

**Authors:** Vivekanand Pandey Vimal, Alexander Sacha Panic, James R. Lackner, Paul DiZio

**Affiliations:** ^1^ Ashton Graybiel Spatial Orientation Laboratory, Brandeis University, Waltham, MA, United States; ^2^ Volen Center for Complex Systems, Brandeis University, Waltham, MA, United States; ^3^ Psychology Department, Brandeis University, Waltham, MA, United States

**Keywords:** spatial disorientation, spaceflight analog, vibrotactile feedback, sensory substitution, dynamic balance, vestibular, human augmentation

## Abstract

Spaceflight can make astronauts susceptible to spatial disorientation which is one of the leading causes of fatal aircraft accidents. In our experiment, blindfolded participants used a joystick to balance themselves while inside a multi-axis rotation device (MARS) in either the vertical or horizontal roll plane. On Day 1, in the vertical roll plane (Earth analog condition) participants could use gravitational cues and therefore had a good sense of their orientation. On Day 2, in the horizontal roll plane (spaceflight analog condition) participants could not use gravitational cues and rapidly became disoriented and showed minimal learning and poor performance. One potential countermeasure for spatial disorientation is vibrotactile feedback that conveys body orientation provided by small vibrating devices applied to the skin. Orientation-dependent vibrotactile feedback provided to one group enhanced performance in the spaceflight condition but the participants reported a conflict between the accurate vibrotactile cues and their erroneous perception of their orientation. Specialized vibrotactile training on Day 1 provided to another group resulted in significantly better learning and performance in the spaceflight analog task with vibrotactile cueing. In this training, participants in the Earth analog condition on Day 1 were required to disengage from the task of aligning with the gravitational vertical encoded by natural vestibular/somatosensory afference and had to align with randomized non-vertical directions of balance signaled by vibrotactile feedback. At the end of Day 2, we deactivated the vibrotactile feedback after both vibration-cued groups had practiced with it in the spaceflight analog condition. They performed as well as the group who did not have any vibrotactile feedback. We conclude that after appropriate training, vibrotactile orientation feedback augments dynamic spatial orientation and does not lead to any negative dependence.

## 1 Introduction

Long duration spaceflight poses many physiological (e.g.,,cardiovascular, bone, muscle, visual and vestibular) and psychological (e.g., isolation, anxiety, depression) stressors on astronauts, making them more susceptible to spatial disorientation. The number of stressors and their potential impact are especially serious during gravitational transitions such as when landing on the surface of a planet or the Moon where astronauts will not have access to familiar gravitational cues and will have undergone prior sensorimotor adaptations to weightless conditions (Shelhamer, 2015; Clément et al., 2020). Spatial disorientation can occur under many circumstances including when there is an inaccurate or attenuated perception of position, motion or attitude (Lackner, 1992; Poisson and Miller, 2014). In addition to the unique stressors of spaceflight such as gravitational transitions and sensory reweighting, some common causes of spatial disorientation shared between spaceflight and aviation include inaccurate sensory information and mismatch between different sensory systems (e.g., the vestibular, visual and somatosensory) (Heinle and Ercoline, 2003). Between 1993 and 2013, spatial disorientation led to 101 deaths. Sixty-five aircraft were lost, resulting in $2.32 billion of damages (Poisson and Miller, 2014). One proposed countermeasure for spatial disorientation is vibrotactile feedback about body orientation provided by small vibrating devices on the skin (Cholewiak et al., 2004). Such feedback has been shown to improve performance (Wenzel and Godfroy-Cooper, 2021) in motion platform control (Bouak et al., 2011), flight simulators (Cardin et al., 2006; Ouyang et al., 2017), helicopter flight (Raj et al., 2000; Lawson and Rupert, 2014), and fixed wing aircraft flight (Rupert, 2000a; Rupert, 2000b). Additional vibrotactile uses include providing cockpit alerts (Salzer et al., 2011), cueing astronaut orientation in the International Space Station (van Erp and van Veen, 2006), performing a nulling task after being rotated in yaw to cause disorientation (van Erp et al., 2006), and a nulling task after returning from spaceflight (Clément et al., 2018).

It remains to be seen how vibrotactile cueing during a dynamic orientation task on transition to a novel background environment will be useful. When disoriented, a pilot’s own internal sensory feedback may be misleading and it is unknown whether vibrotactile feedback will be able to correct this misperception of orientation. If vibrotactile feedback is unable to correct the misperception, it is unknown whether this will create confusion and whether pilots will be able to rely on and trust the vibrotactors during highly stressful and disorienting conditions. Finally, it is unknown whether training can enhance the ability to use vibrotactile feedback to mitigate spatial disorientation.

**FIGURE 1:**
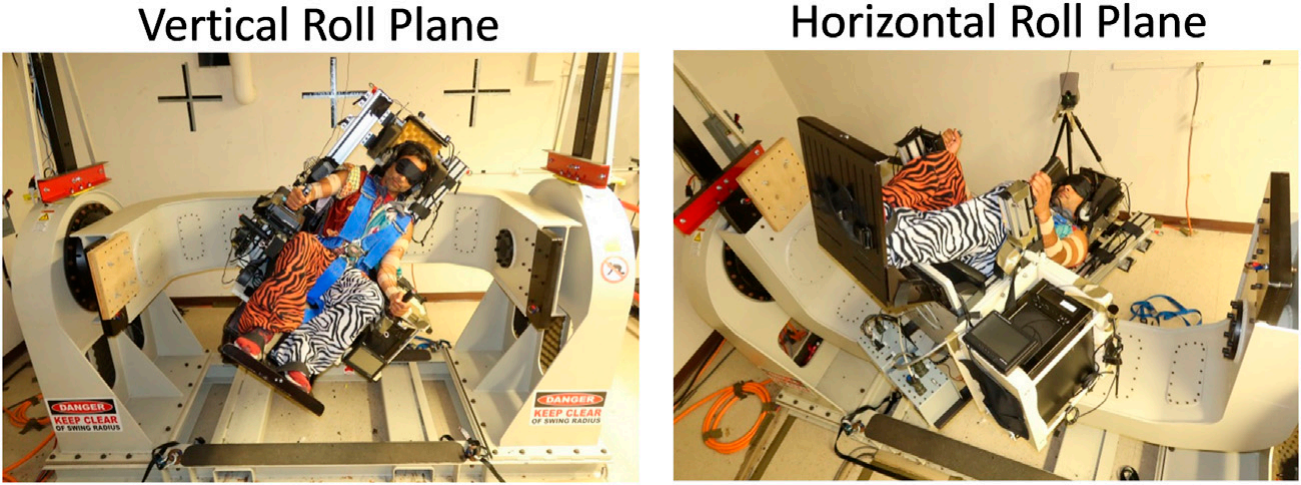
The multi-axis rotation device (MARS) was programmed with inverted pendulum dynamics in the vertical roll plane (left) and the horizontal roll plane (right).

To address these issues we developed a disorienting spaceflight analog task that involved blindfolded participants riding in our Multi-axis Rotation System (MARS) device that was programmed to roll them with inverted pendulum dynamics (Figure 1) (Panic et al., 2015). Participants use a joystick to stabilize themselves around the direction of balance. When the MARS is configured for vertical roll plane motion (Earth analog condition), participants can use gravitational cues detected by their otolith organs and somatosensory forces on their skin to determine their angular position relative to the balance point (Vimal et al., 2016). By contrast, when the MARS is configured for horizontal roll plane motion (spaceflight analog condition), they do not tilt relative to the gravitational vertical. Consequently, they cannot use gravity-dependent otolith and somatosensory shear forces to provide a sense of their angular position in relation to the direction of balance (Panic et al., 2017; Vimal et al., 2017). They only have access to motion cues detected by the semicircular canals and somatosensory receptors. In this condition, participants, as a group, show minimal learning, poor performance, and a very high rate of losing control (Vimal et al., 2017; Vimal et al., 2018). Ninety percent of participants report feeling disoriented and all participants show a characteristic pattern of positional drifting.

In our study, as summarized in Table 1, three groups of participants (OnlyTraining, Vibrotactile, and Vibrotactile + Training) were first exposed to an Earth analog balancing task in the vertical roll plane on Day 1. This was to represent exposure, training and skilled motor learning that astronauts would receive on Earth that are relevant to stabilizing the MARS and learning to use vibrotactile feedback. Then, on Day 2, participants were tested in a spaceflight analog balancing task in the horizontal roll plane. This was to represent the minimal or altered gravitational conditions such as hypo-g or 0 g where an astronaut would need to apply pre-flight 1 g training. Our prior work shows that there is very minimal deterioration in skill even after a gap of several months (Vimal et al., 2019), meaning that skills acquired on Earth could be retained on a journey to Mars or the Moon, however it is unknown how the novel experience of minimal gravitational cues could affect the use of vibrotactile feedback.

**TABLE 1:** Overview of experimental design for each group. The Know/Start/Reset column refers to whether the participant knew the location of the direction of balance (DOB) and whether the MARS started and reset at the DOB. DOB = 0 in the vertical roll plane corresponds to the gravitational vertical and in the horizontal roll plane it corresponds to the same angular orientation of the MARS relative to its gimbal frame (see Figure 1).

Group	Day	Trial	Roll plane orientation	DOB	Know/Start/Reset at DOB?	Vibrotactor
OnlyTraining	1	B1: 1–6	Vertical	0	Yes	No
		B2: 7–10		random	Yes	
		B3: 11–18		random	No	
		B4: 19–20		0	Yes	
	2	B1: 1–4	Horizontal	0	Yes	
		B2: 5–8				
		B3: 9–12				
		B4:13–16				
		B5: 17–20				
Vibrotactile	1	B1: 1–6	Vertical	0	Yes	Yes
		B2: 7–10				
		B3: 11–18				
		B4: 19–20				
	2	B1: 1–4	Horizontal	0	Yes	Yes
		B2: 5–8				
		B3: 9–12				
		B4:13–16				
		B5: 17–20				No
Vibrotactile + Training	1	B1: 1–6	Vertical	0	Yes	Yes
		B2: 7–10		random	Yes	
		B3: 11–18		random	No	
		B4: 19–20		0	Yes	
	2	B1: 1–4	Horizontal	0	Yes	Yes
		B2: 5–8				
		B3: 9–12				
		B4:13–16				
		B5: 17–20				No

To test whether vibrotactile feedback was useful in our disorienting spaceflight analog condition, the Vibrotactile and Vibrotactile + Training groups received vibrotactile feedback that communicated the MARS’ angular deviation from the balance point. We hypothesized that both vibrotactile groups would perform better and show greater learning than the OnlyTraining group who did not receive any vibrotactile feedback. Throughout the experiment, participants reported the level of confusion in their spatial and motion perception and they rated the level of trust they had in the vibrotactile feedback. We hypothesized that they would experience confusion and would mistrust the vibrotactile feedback during their initial exposure to the disorienting spaceflight analog condition.

We further hypothesized that the Vibrotactile group would not perform as well in the spaceflight analog condition as they did in the Earth analog condition, because the exposure in the Earth analog condition might not be sufficient to teach them how to rely on the vibrotactors. This is because participants in the Earth analog condition primarily rely on the cognitively transparent natural gravitationally based cues (Vimal et al., 2017) rather than the effortfully attended synthetic vibrotactile cues. In sensory substitution paradigms, effective use of cueing technology often requires a period of free exploration (active sensing) with the device to build intuitive, effortless associations between the new sensory feedback and the task (Taub and Wolf, 1997; Bach-y-Rita and Kercel, 2003; Bertram and Stafford, 2016; Kim, 2021). In other words, one also needs to create training conditions in which the participants have to rely on the new sensory feedback. To test this we created a specialized training program based on our prior work (Vimal et al., 2019) that required participants to disengage from aligning with otolith/somatosensory cues about gravitational vertical while relying on vibrotactile feedback (for the Vibrotactile + Training group) on Day 1. The OnlyTraining group received the same training on Day 1, however without vibrotactile feedback. We hypothesized that the Vibrotactile + Training group would perform better than the OnlyTraining and Vibrotactile groups in the spaceflight analog condition on Day 2. To determine whether a dependence on the vibrotactors could form, we disengaged the vibrotactors in the last block of the Day 2 spaceflight analog condition, and we hypothesized that performance would worsen however would not be worse than the OnlyTraining group.

## 2 Materials and methods

### 2.1 Participants

30 healthy participants were recruited where 10 participants were in the OnlyTraining group (5 female and 5 male, 25 ± 5 years old), 10 participants were in the Vibrotactile group (5 female, 4 male, and 1 nonbinary, 23 ± 5 years), and 10 participants were in the Vibrotactile + Training group (5 female and 5 male, 24 ± 5 years). All participants signed an informed consent in accordance with the Declaration of Helsinki and the protocol was approved by the Brandeis Institutional Review Board.

### 2.2 Apparatus

We used a Multi-Axis Rotation System (MARS) device that was programmed to roll subjects seated in it with inverted pendulum dynamics, following the equation, $\ddot{\theta }=k_{P}\sin\theta$ , where θ is the angular deviation from the direction of balance (in degrees), and k_P_ is the pendulum constant (Figure 1). We chose a challenging pendulum constant of 600 deg/s^2^ (≈0.52 Hz) so that participants would require substantial exposure to master the task (Vimal et al., 2016; Vimal et al., 2017; Vimal et al., 2018). A velocity increment proportional to the joystick deflection was added to the MARS velocity at every time step (∼50 Hz) and then integrated by a Runge-Kutta RK4 solver to calculate the new MARS angular position and velocity. When participants exceeded the programmed crash boundaries at ±60 deg from the direction of balance, the MARS would stop and reset to the start point after which the trial would resume. We set the angular velocity limit at ±300 deg/s and angular acceleration limit at ±180 deg/s^2^. More details about the control scheme can be found in Panic et al. (2015).

### 2.3 Vibrotactile feedback

To provide vibrotactile feedback (used by the Vibrotactile and Vibrotactile + Training groups), we used elastic bandages to wrap 4 C-2 (manufactured by Engineering Acoustics) vibrotactors on each arm from the shoulder to the wrist at equal intervals. Only the vibrotactors on the side of the MARS deviation from the balance point vibrated. The first vibrotactor (near the shoulder) activated when the MARS deviated beyond 1 deg from the direction of balance, the second beyond 7 deg, the third beyond 15 deg and the fourth (near the wrist) beyond 31 deg. We ran pilot studies to determine the placement and manner in which the vibrotactors operated.

### 2.4 Procedure

Participants were informed that the MARS behaved like an inverted pendulum and were shown a video of the MARS moving without any control input until it reached the crash boundary (±60 deg from the direction of balance) and then reset. They were also shown a video of a person balancing in the MARS around the direction of balance in both the vertical and horizontal roll planes. The OnlyTraining and Vibrotactile + Training groups, both of whom received a specialized training protocol, additionally saw a video of a person balancing the MARS in the vertical roll plane at a direction of balance other than the gravitational vertical. After signing consent forms, participants were secured in the MARS using side torso panels that did not interfere with the arms and vibrotactors, a five-point safety harness, a lap belt, and foot straps. They wore blindfolds, earplugs and a noise cancelling headset that played white noise during the trials. Their heads were supported by a cushioned frame attached to the MARS. A “kill switch” on the left armrest could be pressed to stop the experiment. No participant ever needed to use it. A Logitech Freedom 2.4 cordless joystick was mounted on the right armrest, and the participant used it to control the MARS.

All trials began with an auditory “begin” command. Whenever the MARS reached ±60 deg from the direction of balance (crashed), the joystick was disabled, and participants heard “lost control, resetting” and the MARS automatically reset to the start position at a rate of 5 deg/s. Once at the start position the joystick was enabled and participants heard a “begin” command. Each trial was scheduled to last a total of 100 s of balance time excluding the reset times after crashes, but a trial was stopped when the total elapsed time, including the resets, exceeded 150 s. All three groups took part in 2 experimental sessions on consecutive days. On Day 1 they balanced in the vertical roll plane and on Day 2 they balanced in the horizontal roll plane (Table 1). In each session there were 20 trials, divided into blocks defined in Table 1, with 2-min breaks between blocks. In the final block of Day 2, the vibrotactors were turned off for the Vibrotactile and Vibrotactile + Training groups.

### 2.5 Specialized training program

In our prior work, we showed that a specialized training program could modestly enhance performance in the spaceflight analog condition even without vibrotactile feedback (Vimal et al., 2019). The central idea of this training program was to teach participants to disengage from aligning to the gravitational vertical. We achieved this by randomizing the angular location of the direction of balance while in the vertical roll plane (Earth analog condition). Participants did not know the location of the balance point and had to search for it by focusing on the vibrotactile feedback.

In this experiment, both the OnlyTraining and Vibrotactile + Training groups underwent the same training program on Day 1 in the vertical roll plane similar to Vimal et al. (2019). Before the experiment began, participants were told about strategies that good performers had found useful in prior experiments (Vimal et al., 2019). These strategies included: 1. Use small joystick deflections, 2. Use intermittent joystick deflections, 3. Use smaller joystick deflections when near the direction of balance compared to when far away from it, and 4. Find the direction of balance by switching from falling in one direction to the other. On Day 1, trials one to six they had a direction of balance at 0 deg, that allowed them to learn the paradigm. In trials 7–10, every trial had a different randomized non-zero direction of balance that ranged from 5 to 30 deg. We chose 30 deg as the limit of the direction of balance because the crash boundaries were set 60° from the balance point and during pilot studies participants reported feeling uncomfortable tilting beyond 90 deg. Before the trial began, participants were told the angular location of that direction of balance and the trial started with the MARS at that balance point. When participants lost control, they were reset to that non-vertical balance point. These trials were meant to familiarize participants with balancing at non-zero balance points. Trials 11–18 also had different randomized non-zero directions of balance, but they differed from the previous trials in additionally having randomized start and reset points, which prevented participants from immediately knowing the direction of balance. At the end of the trial, they were told the correct location of the direction of balance. In trials 19–20, participants were told the actual direction of balance, which was 0 deg.

On Day 2, both groups received 20 trials in the horizontal roll plane where the direction of balance was always at 0 deg. We decided against having randomized directions of balance on Day 2 to highlight the difficulty of the task on Day 2 in the horizontal roll plane. Even though the participants knew that the start point, the reset point and direction of balance were always at 0 deg, they still became disoriented quickly as reflected in their performance.

For the Vibrotactile and Vibrotacile + Training groups, the vibrotactors were turned off for the last block; for the OnlyTraining group they were always absent.

### 2.6 Data reduction and analysis

We used a zero-phase 5-pole high-pass Butterworth filter with a cutoff frequency of 5 Hz on the MARS angular position and velocity and joystick deflections all were sampled at 20.7 ± 1.1 msec (approximately 50 Hz). After filtering, we removed data from the reset periods following crashes when participants had no control over the MARS. All of the metrics described below were calculated in each trial, then averaged across all trials in a block, and finally averaged across all participants.

#### 2.6.1 MARS performance

To quantify the positional variability, we calculated standard deviation of MARS angular position (STD_MARS_). The frequency of crashes (Crashes) was calculated by counting the number of crashes in a trial and then dividing by the duration of the trial and then multiplying by 60 to obtain units of min^-1^. The average magnitude of MARS velocity (|Mag|_Vel_) was calculated by taking the mean of the absolute value of the MARS angular velocity. In our prior work (Vimal et al., 2017; Vimal et al., 2019) we found that balance control consists of two dissociable components: position control mediated by gravitational cues sensed by the otoliths and somatosensory receptors, and velocity control mediated by dynamic cues sensed by the semicircular canals and somatosensory receptors.

#### 2.6.2 Joystick command

We calculated the average of the absolute value of joystick deflections (|Mag|_Joy_), which would vary from +1 to −1 when fully deflected. Destabilizing joystick deflections were defined as those that accelerated the MARS away from the direction of balance. The percentage of destabilizing joystick deflections (%Destab) was calculated by dividing the number of data points where the MARS angular position, velocity, and joystick deflection all had the same sign by the total number of data points.

#### 2.6.3 Stabilogram diffusion function (SDF)

**FIGURE 2:**
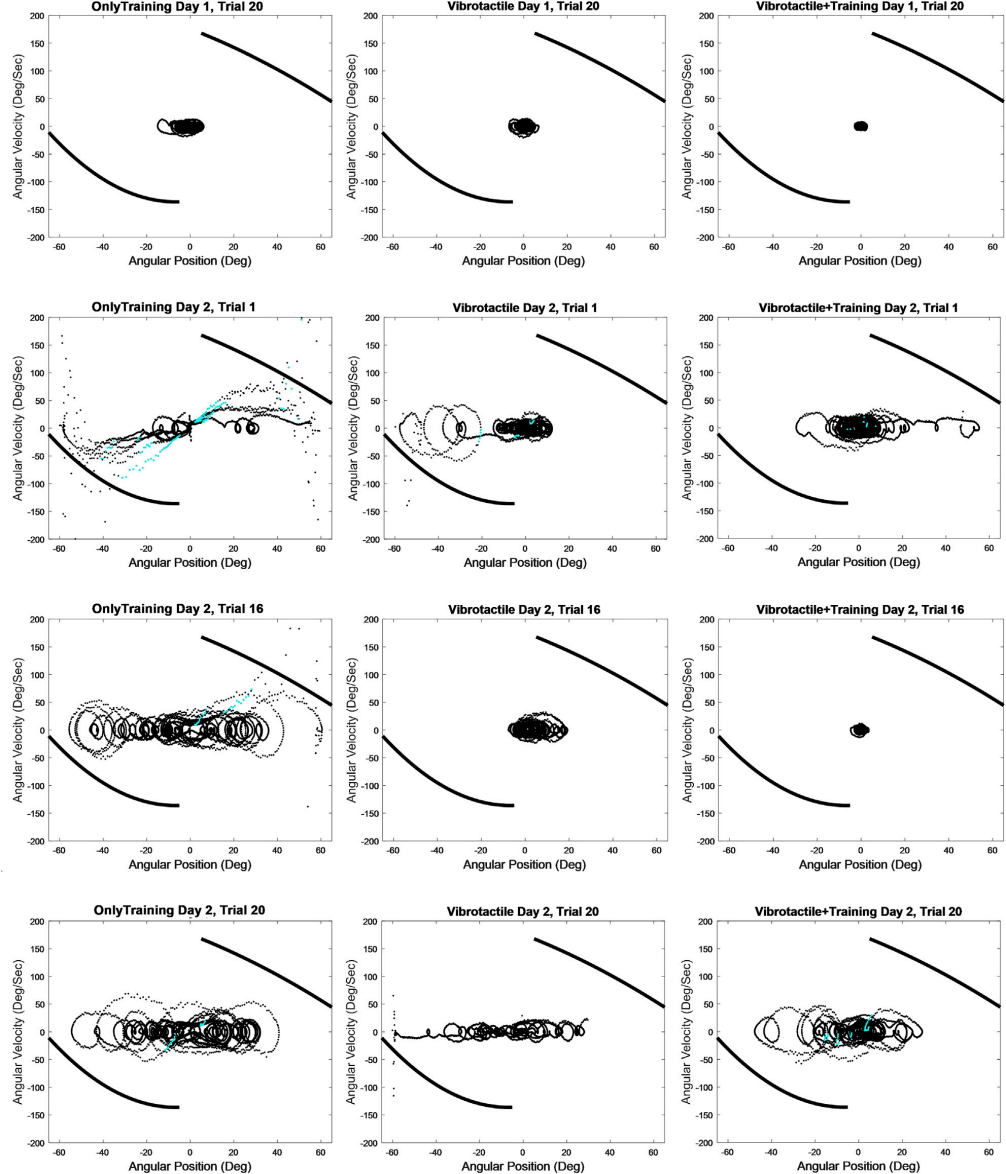
Phase plots of MARS angular velocity against MARS angular position from representative participants from the OnlyTraining (first column), Vibrotactile (second column) and Vibrotactile + Training (third column) groups. The first row is the final trial (trial 20) on Day 1 where participants are in the Earth analog condition (vertical roll plane). The second row is trial 1 on Day 2 where participants are in the spaceflight analog condition (horizontal roll plane). The third row is trial 16 on Day 2 which is the final trial before the vibrotactors are deactivated. The fourth row is trial 20 on Day 2. The thick black curves are empirically determined and represent limits after which joystick deflections cannot prevent crashing. The blue points signify the occurrence of destabilizing joystick.

The SDF quantifies the average stochasticity of a variable (Collins and De Luca, 1993). In several previous publications (Vimal et al., 2016; Vimal et al., 2017; Vimal et al., 2018; Vimal et al., 2019), we have described how we calculate the SDF and its derivative parameters for MARS positional fluctuations following a method published by Collins and colleagues (1993) for discriminating the open and closed-loop control regimes of human bipedal posture. Very short time spans between data samples preclude physiological close-loop control, at an intermediate time span random behavior occurs, and at longer time spans close-loop control becomes evident. The SDF parameter D_L_, the long term diffusion coefficient, quantifies residual positional drift even at long enough time spans where closed-loop control is evident. Positional drifting is important to quantify because it is a characteristic pattern of balancing in the absence of relevant gravitational cues both in the MARS horizontal roll plane (Vimal et al., 2017; Vimal et al., 2019; Vimal et al. 2020) and about the vertical yaw axis (Vimal et al., 2018) as well as in helicopter hovering (Raj et al., 2000; Lawson and Rupert, 2014). Measures such as the STD_MARS_ are unable to capture positional drifting because STD_MARS_ will similarly report large numbers for large oscillations that span a large angular space without drifting as it would for small oscillations with position drifting (e.g., in Figure 2 ‘Vibrotactile Day 2, Trial 20’). D_L_ relates the change in the mean-squared displacement over increasing time windows.

## 3 Results

Our first hypothesis was that the Vibrotactile group would perform better and show greater learning than the OnlyTraining group, which is addressed by the subsections “Vibrotactile vs. OnlyTraining” and “Learning on Day 2”. Typically one would compare the Vibrotactile Group with a control group that did not receive any training. However, in our prior work (Vimal et al., 2019) we found that the specialized training without vibrotactile feedback, which is equivalent to the present OnlyTraining group, modestly enhanced performance in the spaceflight analog condition relative to a control group without the training or vibrotactile feedback. This means the present OnlyTraining group would perform better than a control group that did not have any training. Therefore, any statistical significance showing the Vibrotactile group performed better than our OnlyTraining group would apply to a control group without training with even greater significance. Our second hypothesis was that the Vibrotactile group would not perform as well in the spaceflight analog condition as they did in the Earth analog condition, which is addressed by the subsection “Day 1 into Day 2”. Our third hypothesis was that the Vibrotactile + Training group would perform better than the OnlyTraining and Vibrotactile groups in the spaceflight analog condition on Day 2, which is addressed in the subsections “Vibrotactile + Training vs. OnlyTraining” and “Vibrotactile + Training vs. Vibrotactile”. Our fourth hypothesis was that performance would worsen when the vibrotactors were turned off in the final block of Day 2, which is addressed in the subsections “Vibrotactile vs. OnlyTraining” and “Vibrotactile + Training vs. OnlyTraining”.

The experiment was designed to address questions pertaining to four specific testing periods: 1) the final block on Day 1, which assessed proficiency in the Vertical Roll Plane condition, 2) Block 1 on Day 2, which assessed performance for the first exposure to the Horizontal Roll Plane condition; 3) Block 4 on Day 2, which assessed ultimate proficiency in the Horizontal Roll Plane after practice with or without vibrotactile augmentation, and 4) Block 5 of Day 2 which assessed Horizontal Roll Plane balancing upon withdrawal of vibrotactile augmentation. A MANOVA (SPSS version 28) including all dependent measures with the 4 day/block periods as a within subject factor and the three training groups as a between subjects factor showed that there was a significant main effect of both factors (period: Pillai’s Trace, F (30, 204) = 6.183, *p* < .001; group: Pillai’s Trace, F (26, 30) = 5.705, *p* < .001) as well as a significant period-by-group interaction (Pillai’s Trace, F (78, 426) = 2.246, *p* < .001). Univariate ANOVAs with the same design yielded significant period-by-group interactions for STD_MARS_ (*p* = .0016), Crashes (*p* = .00003), |Mag|_Joy_ (*p* = .023), and %Destab (*p* = .007), and a marginally significant interaction for D_L_ (*p* = .081). Greenhouse-Geisser corrections were applied to the univariate ANOVAs in cases where Mauchly’s Sphericity test was significant. The outcome measures that did not show significant interactions - |Mag|_Vel_ - showed similar trends to the ones that were significant. Given this evidence of interaction between the testing period and the treatment groups, we addressed the specific research questions framed above with paired or independent t-tests with Bonferroni corrections.

### 3.1 Day 1 into Day 2

To determine how performance changed when going from the Earth analog condition (vertical roll plane) to initial exposure to the spaceflight analog condition (horizontal roll plane), we performed paired t-tests between the last block of Day 1 and the first block of Day 2. All groups showed that performance significantly worsened on Day 2 (Table 2). This can also be seen in representative participants in Figure 2, where performance in the second row is much worse (e.g., larger magnitude loops spread across the Position axis) than in the first row. The OnlyTraining group had greater: standard deviation of angular position (STD_MARS_), Crashes, velocity (|Mag|_Vel_), magnitude of joystick deflections (|Mag|_Joy_), destabilizing joystick deflections (%Destab), and long term Diffusion Coefficient (D_L_) that quantifies positional drifting. The Vibrotactile group’s performance worsened and had greater: STD_MARS_, Crashes, |Mag|_Vel_, |Mag|_Joy_, %Destab, and D_L_. The Vibrotactile + Training group’s performance worsened with greater: STD_MARS_, Crashes, |Mag|_Vel_, and |Mag|_Joy_.

**TABLE 2:** Day1 vs. Day 2 performance.

	OnlyTraining (n = 10)		Vibrotactile (n = 10)		Vibrotactile + Training (n = 10)	
Metric	D1B4	D2B1	D1B4	D2B1	D1B4	D2B1
MARS Performance						
STD_MARS_ (deg)	9.08	22.3***	7.2	16.8***	4.7	11.6**
Crashes (min^-1^)	0	0.12***	0	0.048**	0.0006	0.018*
|Mag|_Vel_ (deg/s)	12.8	17.9*	9.4	15.2***	6.5	11.0**
Joystick Commands						
|Mag|_Joy_	0.14	0.22**	0.10	0.18***	0.07	0.13**
%Destab	0.18	1.7**	0.07	0.37*	0.04	0.18
Stabilogram-Diffusion Function						
D_L_ (deg^2^/s)	−1.0	38.7***	0.4	19.2*	0.15	7.5

* represents *p* < 0.05, ** represents *p* < 0.01, *** represents *p* < 0.001.

### 3.2 Learning on Day 2

To determine whether participants showed learning in the spaceflight analog condition, we performed paired t-tests between the first and fourth blocks of Day 2 (Table 3). The OnlyTraining group showed a decrease in the frequency of crashes (Crashes) (*p* = 0.002), and destabilizing joystick deflections (%Destab) (*p* = 0.03). The Vibrotactile group was able to decrease Crashes (*p* = 0.047). The Vibrotactile + Training group showed a decrease in: standard deviation of angular position (STD_MARS_) (*p* = 0.002), Crashes (*p* = 0.048), MARS angular velocity (|Mag|_Vel_) (*p* = 0.009), and magnitude of joystick deflections (|Mag|_Joy_) (*p* = 0.003). This can be seen for representative participants in Figure 2, where performance in the second row is much worse (larger loops that drift across the Position axis) than in the third row for the Vibrotactile + Training group than other groups.

**TABLE 3:** Day 2 performance.

	OnlyTraining (n = 10)			Vibrotactile (n = 10)			Vibrotactile + Training (n = 10)		
Metric	D2B1	D2B4	D2B5	D2B1	D2B4	D2B5	D2B1	D2B4	D2B5
MARS Performance									
STD_MARS_ (deg)	22.3	20.4	20.3	16.8	14.8	19.3	11.6	8.0	15.0
Crashes (min^-1^)	0.12	0.06	0.06	0.048	0.018	0.054	0.018	0.0024	0.03
|Mag|_Vel_ (deg/s)	17.9	18.0	17.8	15.2	14.2	15.8	11.0	8.6	13.4
Joystick Commands									
|Mag|_Joy_	0.22	0.22	0.22	0.18	0.17	0.21	0.13	0.10	0.17
%Destab	1.7	0.91	0.91	0.37	0.25	0.61	0.18	0.060	0.77
Stabilogram-Diffusion Function									
D_L_ (deg^2^/s)	38.7	27.6	38.7	19.2	15.9	19.9	7.5	0.5	18.3

Check the Results section for the statistical significances.

### 3.3 Vibrotactile vs. OnlyTraining

To determine whether there were any differences on Day 2 between the OnlyTraining group and the Vibrotactile group during initial and late exposure to the disorienting horizontal roll condition, we performed an independent t-test between groups for the first block and fourth blocks (Table 3). In Block 1, the Vibrotactile group compared to the OnlyTraining group, had a smaller STD_MARS_ (*p* = 0.008), fewer Crashes (*p* = 0.004), and lower %Destab (*p* = 0.006). In Block 4, the Vibrotactile group compared to the OnlyTraining group had smaller STD_MARS_ (*p* = 0.03), fewer Crashes (*p* = 0.03), and lower %Destab (*p* = 0.04). This can be seen for representative participants in Figure 2, where performance in the first column (OnlyTraining) is much worse (larger loops that drift across the Position axis) than in the second column (Vibrotactile). To determine whether the Vibrotactile group, when deprived of the vibrotactors, performed worse than the OnlyTraining group, we performed an independent t-test between groups for Block 5 and found no significance.

### 3.4 Vibrotactile + Training vs. OnlyTraining

To determine whether there were any differences on Day 2 between the OnlyTraining group and the Vibrotactile + Training group during initial and late exposure to the disorienting horizontal roll condition, we performed an independent t-test between groups for the first block and fourth blocks (Table 3). In Block 1, the Vibrotactile + Training group compared to the OnlyTraining group had smaller STD_MARS_ (*p* = 0.0002), fewer Crashes (*p* < 0.001), lower |Mag|_Vel_ (*p* = 0.01), |Mag|_Joy_ (*p* = 0.004), %Destab (*p* = 0.002), and D_L_ (*p* = 0.002) that quantifies positional drifting. In Block 4, compared to the OnlyTraining group, the Vibrotactile + Training group had smaller STD (*p* = 0.0001), Crashes (*p* = 0.002), |Mag|_Vel_ (*p* = 0.005), |Mag|_Joy_ (*p* = 0.0004), %Destab (*p* = 0.008), and D_L_ (*p* = 0.0009). This can be seen for representative participants in Figure 2, where performance in the first column (OnlyTraining) is much worse (larger loops that drift across the Position axis) than in the third column (Vibrotactile + Training). To determine whether the Vibrotactile group with deactivated vibrotactors was worse than the OnlyTraining group, we performed an independent t-test between groups for Block 5 and found no significance.

### 3.5 Vibrotactile + Training vs. Vibrotactile

To identify any group differences between the Vibrotactile + Training and Vibrotactile groups during initial and late exposure to the disorienting condition on Day 2, we performed an independent t-test between groups for the first block and fourth blocks (Table 3). In Block 1, the Vibrotactile + Training group compared to the Vibrotactile group, had lower |Mag|_Joy_ (*p* = 0.04). In Block 4, the Vibrotactile + Training group compared to the Vibrotactile group had smaller: STD_MARS_ (*p* = 0.009), |Mag|_Vel_ (*p* = 0.01), |Mag|_Joy_ (*p* = 0.006), and %Destab (*p* = 0.04). This can be seen for representative participants in Figure 2, where performance in the second column (Vibrotactile) is worse (larger loops) than in the second column (Vibrotactile + Training).

### 3.6 Survey questions

These questions had been asked after Block 1 on Day 1, after Block 4 on Day 1, after trial 1 on Day 2 and after Block 4 on Day 2. To determine any change in values, we performed paired t-tests.

#### 3.6.1 Spatial and motion perception

Participants were asked to indicate whether they were confused about where they were in relation to the balance point, 1 meant they were not confused and 10 meant that they were very confused. When using a paired t-test to compare the responses from the end of Day 1 and the responses after trial 1 on Day 2, the OnlyTraining group had an increase from 1.8 to 7.8 (p = 2e-6), the Vibrotactile group had an increase from 1.7 to 6.8 (p = 3e-4), and the Vibrotactile + Training group had an increase from 1.4 to 6.6 (p = 1e-4). Using a paired t-test to compare the responses between trial 1 on Day 2 and the end of Day 2, only the OnlyTraining group showed a statistically significant decrease from 7.8 to 5.6 (*p* = 0.01).

At the end of Block 2, participants were asked whether their ability to detect motion decreased in the horizontal roll plane compared to the vertical roll plane on a 10 point scale. The OnlyTraining group reported a 6.3 point decrease (p = 3e-6), the Vibrotactile group had a 6.4 point decrease (*p* = 1.3e-6), and the Vibrotactile + Training group had a 4.4 point decrease (*p* = 0.002).

#### 3.6.2 Participant experience with vibrotactors

The Vibrotactile and Vibrotactile + Training groups were asked to report the trust they had that the vibrotactors were accurately indicating the location of the balance point. For both groups, there was no statistical change across Day 1, or the transition to Day 2 or across Day 2. On average, trust was rated at 84% for the Vibrotactor group and 92% for the Vibrotactile + Training group. When asked about the usefulness of vibrotactors (−10: They are distracting and make me perform worse; 0: They neither help nor hurt; 10: They are very useful and I couldn’t balance without them). The Vibrotactile group did not report a change in the usefulness across Day 1 or in the transition from Day 1 into Day 2 (average of 7.1) however they did report an increase between the first trial of Day 2 and the final block from 7.2 to 8.8. The Vibrotactile + Training group reported an increase in usefulness across Day 1 from 7.8 to 9.1 (*p* = 0.03) but no change in the transition to Day 2 and across Day 2 where it was on average 9.0.

When asked whether they felt the vibrotactors served as an extension of their body, where 0 meant the vibrotactors did not feel like a part of their body and 10 meant that it felt like an extension of their body, the Vibrotactile group did not report an increase on Day 1 (average of 7.1) or in the transition to Day 2 or across Day 2 (average of 7.3). The Vibrotactile + Training group showed an increase from 5.5 to 6.7 (*p* = 0.05) and then no change across the days and an increase on Day 2 from 5.9 to 7.3 (*p* = 0.03).

After the first block on Day 2, participants were asked whether they felt a conflict between their inner sense of the location of the balance point and what the vibrotactors were indicating. Ninety percent of participants reported that their inner sense of their angular position was different than what the vibrotactors were indicating.

## 4 Discussion

### 4.1 Does vibrotactile feedback help in the spaceflight analog condition?

The Vibrotactile group performed better than the OnlyTraining group in the beginning (Block 1) of Day 2 when they were in the spaceflight analog condition (Figure 2; Table 3). The OnlyTraining group had 2.5 times more frequent Crashes, larger positional variability around the balance point (STD_MARS_), and 4.6 times more frequent destabilizing joystick deflections (%Destab). These findings show that vibrotactile feedback can enhance stabilization performance in a spaceflight analog condition where participants cannot rely on gravitational cues and where they otherwise become spatially disoriented.

### 4.2 Does vibrotactile feedback in the spaceflight analog condition restore performance to the level of the earth analog condition?

When comparing the final block on Day 1 in the Earth analog condition (vertical roll plane) where participants could use gravitational cues, to the first block on Day 2 of the spaceflight analog condition (horizontal roll plane), we found that all groups performed significantly worse across a majority of the metrics (Table 2). Why were both vibrotactile groups in the spaceflight analog condition unable to completely recover performance? When asked to report their magnitude of confusion about their self-orientation, all groups reported an average of 300%–370% increase in their confusion between Day 1 (Earth analog) and Day 2 (spaceflight analog). When questioned at the end of Block 1 on Day 2, 90% of vibrotactile users from both tactor groups reported that their perception of self-orientation did not match what the vibrotactors were indicating. In other words, when the participants were in the spaceflight analog condition (horizontal roll plane) and experienced disorientation, the vibrotactors led to a feeling of confusion and conflict, and participants had to determine whether to follow their inner sense of orientation or use the vibrotactors. These findings show, for the first time, that during disorienting and high stress conditions where each participant’s perception of their orientation can be vastly different (Vimal et al., 2022) and where very large perceptual errors occur, vibrotactile feedback may not be immediately useful.

### 4.3 Does a specialized training program lead to better usage of vibrotactile feedback in the spaceflight analog condition?

Our prior work (Panic et al., 2015; Vimal et al., 2017) showed that there are two dissociable components to balance control: 1. alignment to gravitational vertical, and 2. dynamic stabilization. In the Earth analog condition (vertical roll plane), participants primarily rely on gravitational cues to align to the gravitational vertical. It is likely that on Day 1 participants in the Vibrotactile group primarily focused on the gravitational cues provided by their otoliths and somatosensory receptors and paid little attention to the vibrotactors, which provided less intuitive redundant cues. By contrast, on Day 1 the Vibrotactile + Training group’s task required them to disengage from relying on gravitational cues while relying on vibrotactile and motion cues to successfully perform the task. We had achieved this by randomizing the location of non-vertical balance points in the Earth analog vertical roll condition. Participants did not know the locations of the balance points and had to search for them and then stabilize around them. For example, when the balance point was set at 10° from the gravitational vertical, all of the vibrotactors were off and the more a participant deviated from 10°, the greater number of vibrotactors would turn on. These participants had to focus on the vibrotactors to find the balance point while disengaging from aligning with the gravitational vertical.

Compared to the OnlyTraining group who received the same training, the Vibrotactile + Training group performed significantly better than the Vibrotactile group in the first block, across a greater number of metrics and with a greater magnitude of significance (Results and Table 3). They had better positional (STD_MARS_) and velocity based control (|Mag|_Vel_), few crashes (Crashes), better joystick control (|Mag|_Joy_, %Destab) and less positional drifting (D_L_) which is a characteristic feature of balancing in the spaceflight condition likely caused by poor angular path integration in the absence of gravitational cues. The Vibrotactile + Training group also performed statistically better when compared to the Vibrotactile group. These results show that the training program was effective for the Vibrotactile + Training group and resulted in significantly better performance in early exposure to the disorienting condition (Block 1). Nevertheless, in Block 1 of the spaceflight analog condition, the Vibrotactile + Training group did not perform as well as they had in the final block of the Earth analog condition on Day 1 (Figure 2; Table 2) and showed elevated levels of crashing. Importantly, 90% of both the Vibrotactile and Vibrotactile + Training groups reported confusion and a conflict in which they perceived their orientation to be different from what the vibrotactors were indicating. Therefore the training did not reduce the feeling of conflict but it did help the participants overcome this conflict.

### 4.4 Do participants learn with greater exposure to the spaceflight analog condition?

While participants were informed that they would be in the horizontal roll plane on the second day, they were not told that they may experience spatial disorientation. Would participants perform better, after Block 1, once they knew that they were disoriented and that their internal perception of orientation was incorrect? Surprisingly, the Vibrotactile group showed minimal learning on Day 2 (Table 3; Figure 2), only learning to reduce the frequency of Crashes with a marginal significance (*p* = 0.047). By contrast, the Vibrotactile + Training group showed significant learning by improving positional, velocity and joystick control (STD_MARS_, Mag|_Vel_, |Mag|_Joy_) and reducing the number of crashes (Crashes). By the fourth block on Day 2, the difference between the Vibrotactile + Training and the Vibrotactile group significantly widened on most measures (STD_MARS_, |Mag|_Vel_, |Mag|_Joy_, and %Destab).

Both vibrotactile groups by the end of trial 1 on Day 2 expressed awareness that they were disoriented and that a conflict existed between the perception of their orientation and what the vibrotactors were indicating. There was no statistical difference in their ratings of trust of the vibrotactile feedback between the Earth analog condition and the spaceflight analog condition (84%–92% trust). It is important to emphasize that the Vibrotactile group had both knowledge that they were disoriented and high levels of trust in the vibrotactile feedback and yet they were unable to rely on the vibrotactile feedback. Why was the Vibrotactile group unable to continue learning even though they knew that they were disoriented and trusted the vibrotactile feedback? One possibility is that they were unable to build an association between their orientation and the vibrotactile feedback. In the sensory substitution literature, effective training often requires free exploration (active sensing) to build a strong association with the new sensory feedback device (Bach-y-Rita and Kercel, 2003; Bertram and Stafford, 2016). Our Day 1 exposure allowed the Vibrotactile group to have this free exploration with the vibrotactors, however they most likely relied on the gravitational cues to complete the task and not the vibrotactile cues. This is reflected in their responses to the survey, where they did not report any increase in the usefulness of the vibrotactors across the trials nor did they feel like the device became an extension of themselves on Day 1 or 2, whereas the Vibrotactile + Training group did show a significant increase in their report of usefulness by the end of Day 1 and an increase in the feeling that the device became an extension of themselves on Day 2. These results suggest that to build association between human and device, especially where one is trained and tested in different environments, one must give participants a training condition where the task demands that they exclusively use the device.

### 4.5 Does vibrotactile feedback create dependence?

In the final block of Day 2 in the spaceflight analog condition, we deactivated the vibrotactors to determine whether performance would become significantly worse than for the OnlyTraining group. If so, this would signify that the vibrotactors created a negative dependence. We found that in the final block (Table 3; Figure 2), both the Vibrotactile and the Vibrotactile + Training groups did not perform worse than the OnlyTraining group and instead showed a slight improvement by having lower mean-squared displacements. These results indicate that the vibrotactors did not create a negative dependence and instead helped the participants acquire a similar level of improvement and learning as the OnlyTraining group who showed significant learning, across blocks, as reflected in decreasing the frequency of crashes and the percentage of destabilizing joystick deflections.

### 4.6 Why are the vibrotactile cues unable to correct participants’ perception?

In the spaceflight analog condition, participants cannot use the gravitational cues that they normally would use to stabilize around the direction of balance. While participants in principle should be able to integrate the velocity signals from the semicircular canals to obtain a sense of their angular position, our prior work shows that they lose awareness of their ongoing position within seconds (Vimal et al., 2022) which leads to a characteristic pattern of positional drifting (measured by D_L_) both in the MARS horizontal roll plane (Vimal et al., 2017; Vimal et al., 2019; Vimal et al., 2020) and about the vertical yaw axis (Vimal et al., 2018). On Earth, the error accumulation from the path integration is calibrated by gravitational cues detected by the otolith organs and somatosensory receptors which is absent in the spaceflight analog condition. We tried to replace these missing positional cues with accurate graded positional information from the vibrotactors, however both vibrotactile groups were unable to raise performance in their spaceflight analog condition to that of the Earth analog. One possibility is that they may require much more exposure in the vertical roll plane. The sensory substitution literature shows that, depending on the device, it can take from 25 min to a year of intensive training before proficient performance is obtained using a new sensory feedback device (Bertram and Stafford, 2016). The majority of the sensory substitution literature is based on training and testing the device in the same environment. In our experiment, the training occurred in an Earth analog condition and the testing occurred in a novel spaceflight analog condition. Our future work will determine whether simple exposure to vibrotactor cueing for more extended periods of time coupled with our specialized training could result in better performance. Another possibility is that vibrotactile feedback as used here may not be biologically compelling in conveying “urgency” (Arrabito et al., 2019). Participants reported that vibrotactile cues in the horizontal roll plane did not elicit the urgency and “danger” of being near the crash boundaries that was felt in the vertical roll plane with gravitational and somatosensory cues. It is possible that using more biologically relevant cues such as pressure cues in addition to vibrotactors would require much less training.

We chose to encode only angular position in the vibrotactile feedback because detection of angular acceleration by the semicircular canals should have remained unchanged between the vertical and horizontal roll planes. However, on average, participants reported more than a 50% reduction in their ability to sense motion. One reason for this may be that neural circuits that usually respond to both otolith and semicircular canal signals may need to reweight their contributions to put more emphasis on the semi-circular canal signals (Hupfeld et al., 2022). In future work we will determine whether adding a velocity component to the vibrotactile feedback will enhance performance.

## 5 Conclusion

We found that vibrotactile feedback enhanced stabilization performance, without creating a negative dependence, in a disorienting spaceflight analog condition where participants could not rely on gravitational cues. However, the vibrotactile feedback in the spaceflight condition did not restore performance to the Earth analog condition because participants experienced a feeling of conflict between their perception of orientation and their actual orientation indicated by the vibrotactors. Knowledge of being disoriented and high levels of trust in the vibrotactile feedback were not enough to allow continued learning in the spaceflight analog condition, suggesting that in stressful disorienting conditions that demand fast reactions, trust and knowledge are not enough to ensure reliance on vibrotactile feedback. Instead, a training program, in the Earth analog condition, where participants had to disengage from aligning with gravitational vertical while focusing on vibrotactile feedback was needed to acquire much better performance and sustained learning in the spaceflight analog condition. The training program did not reduce the feeling of conflict however it allowed participants to overcome it.

Our work contributes to a broad effort to enable space exploration with vibrotactile feedback. For example, it could be useful for recognizing alerts (Salzer, Oron-Gilad, Ronen and Parmet, 2011) such as during flight or egress. Vibrotactors could be used as a sensory augmentation device, enhancing performance of manual control tasks (Clément et al., 2018) such as during extravehicular activity (Bakke and Fairburn, 2019). After landing on the surface of a planet or the Moon or returning to Earth, vibrotactile feedback could likely help with postural instability (Sienko et al., 2013; Wall, 2010), and later on, with navigation while exploring the surface (Erp et al., 2005). During flight, vibrotactile feedback could be useful as an aid for maneuvers like sustained hovering (Raj et al., 2000; Lawson and Rupert, 2014). Our research shows that vibrotactile feedback will also be a useful countermeasure for spatial disorientation however will require specialized training.

Finally, our work extends the sensory substitution literature (Bach-y-Rita and Kercel, 2003; Bertram and Stafford, 2016) where individuals are usually trained and tested to use a feedback device in the same environment. In our paradigm, individuals are trained to use vibrotactors in one environment (Earth analog) and then tested in a novel environment (spaceflight analog). We find that effective use of the vibrotactors requires not only free exploration (active sensing) but also specialized training that teaches individuals to disengage from one sense while focusing on the vibrotactile feedback. This could be relevant for other work where body systems or environment can change significantly, such as in rehabilitation (Alahakone and Senanayake, 2009; Wall, 2010; Sienko et al., 2013; De Angelis et al., 2021), sports (van Breda et al., 2017), virtual, augmented and mixed realities (Islam and Lim, 2022), human enhancement and augmentation (Raisamo et al., 2019).

## Data Availability

The raw data supporting the conclusion of this article will be made available by the authors, without undue reservation.
